# Airway Management in the Emergency Department During Coronavirus Disease (COVID-19)

**DOI:** 10.15388/Amed.2020.27.2.1

**Published:** 2020-12-21

**Authors:** Sohil Pothiawala

**Affiliations:** Department of Emergency Medicine, Woodlands Health Campus, Singapore ORCID id: https://orcid.org/0000-0002-4789-4326

**Keywords:** airway, emergency department, emerging viral disease

## Abstract

**Summary. **Front-line health-care workers in the Emergency Department (ED) are at an increased risk of infection during the airway management of patients with known or suspected Emerging Viral Diseases (EVD) like Coronavirus Disease 2019 (COVID-19). The primary route for transmission of the virus from an infected patient to the ED staff is due to aerosolized droplets, and the transmission risk is high despite wearing adequate Personal Protective Equipment (PPE). There are limited evidence-based guidelines for airway management during these viral infections, especially with a focus on the principles of airway management in a busy, fast-paced ED. This article provides an overview of the principles of airway management in suspected or confirmed EVD patients, including COVID-19, particularly in the context of ED, and also considering strategies in resource limited setting. These principles should be adapted to suit your local department and hospital policy on airway management as well as national guidelines.

## Abstract (lt)

**Kvėpavimo takų palaikymas skubiosios pagalbos skyriuje sergant ar įtariant koronavirusą (COVID-19):**

**Santrauka. **Priešakinėse linijose dirbantiems greitosios medicinos pagalbos (GP) skyriaus darbuotojams, palaikantiems pacientų, kuriems nustatyta ar įtariama nauja virusinė liga (angl. *Emerging Viral Diseases*), pavyzdžiui, koronavirusas 2019 (COVID-19), kvėpavimo takus, kyla didesnė užsikrėtimo rizika. Sergantis pacientas dažniausiai GP darbuotojus užkrečia lašeliniu būdu per orą ir, nepaisant to, kad dėvima apsauginė apranga, užkrėtimo rizika yra didelė. Trūksta įrodymais pagrįstų gairių, kaip palaikyti pacientų, sergančių tokiomis virusinėmis infekcijomis, kvėpavimo takus, ypač tokių, kurios aprašytų kvėpavimo takų palaikymą dinamiškame GP skyriuje. Šiame straipsnyje apžvelgiami pacientų, kuriems įtariama ar patvirtinta nauja virusinė liga, įskaitant COVID-19, kvėpavimo takų palaikymo principai, ypač GP skyriuje, ir atsižvelgiama į strategijas, taikomas tada, kai ištekliai yra riboti. Šie principai turėtų būti pritaikyti skyrių ir ligonių kvėpavimo takų palaikymo politikoje ir nacionalinėse gairėse.

**Raktažodžiai: **kvėpavimo takai, greitosios pagalbos skyrius, nauja virusinė liga.

## Introduction

Over the last 20 years, Emerging Viral Diseases (EVD), such as the Severe Acute Respiratory Syndrome Coronavirus (SARS-CoV-1) in 2003, H1N1 influenza in 2009, Middle East Respiratory Syndrome Coronavirus (MERS-CoV) in 2012, Ebola in 2014 and the ongoing pandemic of Severe Acute Respiratory Syndrome Coronavirus-2 (SARS-CoV-2) have threatened humanity and affected the healthcare systems world-wide. The basic demographic information regarding the infectivity rate and case fatality rate of these EVDs is compared in Table 1 [1, 2, 3, 4, 5].

**Table 1.** The basic demographic information comparing the Emerging Viral Diseases in the last 2 decades

	SARS-CoV-1	H1N1	MERS	Ebola	SARS-CoV-2
**Virus emergence**	November 2002, Foshan, China	April 2009, California, USA	April 2012, Zarqa, Jordan	December 2013, Guinea, West Africa	December 2019, Wuhan, China
**Status of pandemic**	Ended	Ended	Ended	Sporadic cases	Ongoing
**Total number of infected cases**	8098	60.8 million	2442	28,652	33,178,019*
**Total number of attributed deaths**	774	151,700-575,400	842	11,325	998,784*
**Case Fatality Rate**	9.5%	0.001% - 0.007%	34%	39.5%	3%

* Data updated till 28^th^ September 2020

Transmission of SARS-CoV-2 can occur when a person is in close contact (approximately 2 meters), and also through respiratory droplets and aerosols. This poses a considerable risk of exposure to the healthcare staff treating these patients in the Emergency Department (ED). This risk is enhanced, especially during airway management in a busy, fast-paced setting of the ED. Aerosol-generating procedures are defined as procedures that are more likely to produce a high concentration of infectious airborne particles (aerosols), than coughing, sneezing, talking, or breathing, and thus increase the risk of transmission of infections. [6] The highest viral load of SARS-CoV-2 has been found in the sputum and upper airway secretions of COVID-19 patients, thus any of these aerosol-generating procedures lead to an increased risk of transmission [7].

Studies have reported an increased risk for SARS-CoV-2 infection among front-line health-care workers in direct contact with patients with COVID-19, despite using adequate Personal Protective Equipment (PPE). But the risk was especially high among those who had inadequate PPE or who were required to reuse PPE [8]. This highlights the importance that emergency physicians and nurses must be aware of this increased risk of exposure when managing these patients. Generic international guidelines and recommendations are available for the risk assessment and management of health-care workers caring for patients with SARS-CoV-2 [9, 10, 11].

There are no guidelines developed specifically for the staff working in the rapidly changing environment such as the ED. Thus, it is vital to device strategies and workflows to protect the health and safety of ED staff who are involved in managing the rapidly deteriorating airway of patients with EVDs like COVID-19. 

## Discussion

The most common manifestation of COVID-19 illness is acute respiratory infection, with fever, dyspnea and cough being the predominant symptoms. As the disease progresses, the patients develop increasing breathlessness, with decreased oxygen saturation, likely secondary to underlying viral pneumonia. Airway interventions in ED are mainly required to support these breathless patients, and they range from giving oxygen through a non-rebreather mask, nebulization, Non-Invasive Ventilation (NIV) and even tracheal intubation to optimize and support ventilation. 

Among the aerosol-generating procedures, endotracheal intubation carries the highest risk of transmission, followed by other procedures like nebulization, open suctioning of airways, non-invasive mechanical ventilation, trachesotomy and bronchoscopy [6]. A systematic literature review and meta-analysis evaluating the transmission of SARS-CoV-1 to health care personnel exposed to aerosol-generating procedures found an increased odds ratio of 6.6 and a 10% absolute increase in risk of transmission to health care worker performing intubation [12]. Table 2, derived from the same study, compares the risks of SARS transmission to healthcare staff exposed to various aerosol generating procedures versus those who were not exposed.

**Table 2.** Risk of SARS-CoV-1 transmission to healthcare staff exposed to aerosol generating procedures compared to those who were not exposed

Aerosol-Generating Procedure	Odds Ratio (95% CI)
Manipulation of oxygen mask	4.6 (0.6 – 32.5)
Nebulization	0.9 (0.1 – 13.6)
Non-Invasive Ventilation	3.1 (1.4 – 6.8)
Tracheal Intubation	6.6 (2.3 – 18.9)
Suction before intubation	3.5 (0.5 – 24.6)
Suction after intubation	1.3 (0.5 – 3.4)
Mechanical ventilation after intubation	0.9 (0.4 – 2.0)
Tracheostomy	4.2 (1.5 – 11.5)

### Oxygen-delivery devices

High-flow airway devices which provide 6 L/min or more of oxygen are preferably avoided if an isolation room is not available to manage the patient [13]. It is also recommended to avoid nebulization, and use Metered Dose Inhaler (MDI) with spacer to reduce the risk of aerosol transmission. There is also some controversy regarding the use of NIV and High-Flow Nasal Oxygenation (HFNO). Both are aerosol-generating procedures and hence potentially increase the risk of viral transmission. Recent study recommends HFNO and NIV to have limited aerosol dispersion when the cannula and/or mask is properly fitted and also by ensuring that the attending staff is wearing the recommended PPE [14, 15].

### Personal Protective Equipment (PPE)

Along with other Infection Prevention and Control measures, PPE constitutes an important but only one part of the entire system to prevent infection of HCWs during patient care, especially airway management. It should be used when managing all COVID-19 patients. Strict adherence to PPE [powered air purifying respirator (PAPR) or N95 mask, goggles, face shield, gown, double gloves pulled over end of gown sleeves] is needed [16]. PAPR is preferred during tracheal intubation, but in a limited resource setting where PAPR is not available, the team members should at least wear an N95 mask during the entire process. Appropriate care should be taken by all staff during the doffing and disposal of PPE after intubation. It should be appropriately disposed after use to reduce the risk of transmission due to reuse, as noted earlier. Staff should be routinely trained on the appropriate use of PPE to ensure their as well as patient safety.

### Tracheal intubation

About 5% of suspected or confirmed COVID-19 patients will develop severe hypoxia or Acute Respiratory Distress Syndrome (ARDS), necessitating tracheal intubation in the ED. There are also patients brought in cardiac arrest to the ED, and it may be difficult to ascertain whether they have underlying COVID-19 disease. Hence, the ED team has to be very vigilant and ensure adequate precautions when managing this group of patients who are critically ill and need tracheal intubation in the ED. Most EDs use an intubation checklist while managing airway in a critically ill patient, hence the use of a checklist that has been modified for use in the Emerging Viral Disease (EVD) patients should be adopted [17].

**Figure 1.**
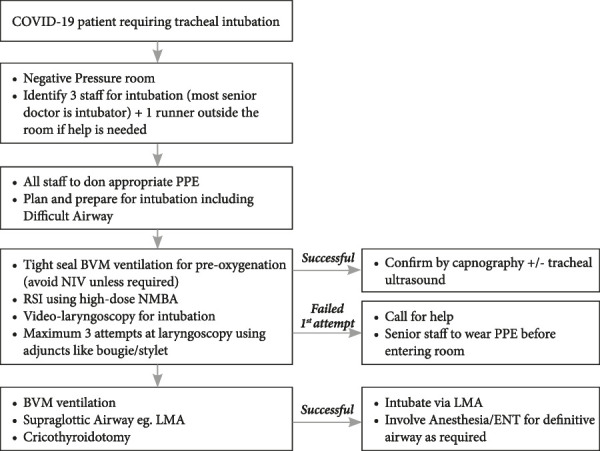
Proposed workflow for tracheal intubation in the emergency department during COVID-19 BVM – Bag Valve Mask; NMBA – Neuro Muscular Blocking Agents; LMA – Laryngeal Mask Airway

The proposed workflow for tracheal intubation in the ED during COVID-19 is shown in Figure 1. These patients should be managed in a negative pressure isolation room. The airway team should be limited to a maximum of 3 people, the most senior and experienced physician, an experienced nurse, and a third person to give drugs and monitor and chart vital signs. Another nurse, stationed outside the room, should monitor the overall situation and call for additional help if required. It is preferable to use single-use equipment [18], but in a setting which precludes its use, reusable equipment may be used, which will then require an appropriate decontamination after the procedure. Ensure a tight seal of the Bag Valve Mask (BVM) during pre-oxygenation. Avoid using Non-Invasive Positive Pressure Ventilation (NIPPV) for pre-oxygenation due to an increased risk of aerosolization. 

Tracheal intubation should be performed using Rapid Sequence Intubation (RSI) with high-dose neuromuscular blocking agents (NMBA) to suppress cough during intubation, thus reducing the risk of aerosolization [19]. Video laryngoscopy with a separate screen is preferred over direct laryngoscopy, as it aids in distancing the operator’s face from the patient’s mouth during intubation. Where a video-laryngoscope is not used or is not available, direct laryngoscopy using MacIntosh blade and a bougie should be used. The intubator should be cautious when removing the bougie or stylet after intubation, to avoid inadvertent spraying of secretions on other members of the intubation team. A transparent plastic sheet or acrylic aerosol box can be placed over the patient’s head and upper thorax during intubation to reduce contamination by preventing droplet spread. But it is important to also consider the limitations of using these substitutes, and they must be used as an additional aid to using standard PPE and adopting universal precautions [20]. All the instruments used during the procedure must be secured in a labeled, transparent bag for disposal and/or decontamination.

### Post-intubation care and mechanical ventilation

Post-intubation lung auscultation may be difficult wearing PPE including PAPR. Watch for equal bilateral chest wall expansion and use continuous end tidal waveform capnography. Use of ultrasound is another means to confirm placement of the endotracheal tube. Prior to commencing mechanical ventilation, cuff of the endotracheal tube should be inflated to avoid any air leak. The initial ventilation settings of tidal volume of 4–8 ml/kg of predicted body weight, respiratory rate of 16-20 breaths/min, PEEP of 5 cm H_2_O and plateau pressures <30 cm H_2_O can be subsequently titrated as necessary. Detailed ventilation modes and strategies are beyond the scope of this article. Use a high-efficiency particulate air (HEPA) filter on the expiratory limb of the mechanical ventilator before initiating ventilation. Closed tracheal suction must be used wherever available. Also, the staff performing the suctioning should wear adequate PPE. Similar precautions should be adhered to in cases of accidental dislodgement of the endotracheal tube. The single-use equipment used during the procedure should be disposed safely, and staff should also ensure appropriate decontamination of the reusable equipment.

### Role of simulation in pandemic preparedness

EDs also play a pivotal role in emergency preparedness to better handle these pandemics. Every ED should organize team based in-situ simulation pertaining to airway management for EVDs, as this will help to train staff appropriately as well as help identify gaps that can otherwise go unnoticed. This will also enable healthcare staff to be better prepared to face challenges during emergent patient airway management situations. 

## Conclusion

Among the aerosol-generating procedures routinely performed in the ED, endotracheal intubation exposes the healthcare worker to the highest risk of procedure-related transmission of COVID-19 and other infectious diseases. The principles of airway management described in this report aim to protect the health and safety of emergency physicians and nurses who are involved in managing the rapidly deteriorating airway of patients during emerging viral diseases like COVID-19. Front-line healthcare workers need to ensure appropriate use of PPE and adherence to all other infection control measures to reduce this risk of transmission. A strategic approach based on the rigorous application of these principles of airway management should be adapted to suit your local department and hospital policy on airway management, while also ensuring adherence to national guidelines.

## Meeting

This manuscript has not been presented in abstract form at any meeting.
